# Machine Learning Model Based on Prognostic Nutritional Index for Predicting Long‐Term Outcomes in Patients With HCC Undergoing Ablation

**DOI:** 10.1002/cam4.70344

**Published:** 2024-10-23

**Authors:** Nan Zhang, Ke Lin, Bin Qiao, Liwei Yan, Dongdong Jin, Daopeng Yang, Yue Yang, Xiaohua Xie, Xiaoyan Xie, Bowen Zhuang

**Affiliations:** ^1^ Division of Interventional Ultrasound, Department of Medical Ultrasonics Institute of Diagnostic and Interventional Ultrasound, The First Affiliated Hospital of Sun Yat‐sen University Guangzhou Guangdong China; ^2^ Department of Microsurgery and Orthopedic Trauma The First Affiliated Hospital of Sun Yat‐sen University Guangzhou Guangdong China

**Keywords:** hepatocellular carcinoma, local ablation, machine learning model, prognosis, prognostic nutritional index

## Abstract

**Aims:**

To develop multiple machine learning (ML) models based on the prognostic nutritional index (PNI) and determine the optimal model for predicting long‐term survival outcomes in hepatocellular carcinoma (HCC) patients after local ablation.

**Methods:**

From January 2009 to December 2019, we analyzed data from 848 primary HCC patients who underwent local ablation. ML models were constructed and evaluated using the concordance index (C‐index), concordance‐discordance area under curve (C/D AUC), and Brier scores. The optimal ML model was interpreted using the partial dependence plot (PDP) and SHapley Additive exPlanations (SHAP) framework. Additionally, the prognostic performance of our model was compared with other models.

**Results:**

Alkaline phosphatase, preoperation alpha‐fetoprotein level, PNI, tumor number, and tumor size were identified as independent prognostic factors for ML model construction. Among the 19 ML algorithms tested, the Aorsf model showed superior performance in both the training cohort (C/D AUC: 0.733; C‐index: 0.736; Brier score: 0.133) and validation cohort (C/D AUC: 0.713; C‐index: 0.793; Brier score: 0.117). The time‐dependent AUC of the Aorsf model for predicting overall survival was as follows: 1‐, 3‐, 5‐, 7‐, and 9‐year were 0.828, 0.765, 0.781, 0.817, and 0.812 in the training cohort, 0.846, 0.859, 0.824, 0.845, and 0.874 in the validation cohort, respectively. The PDP and SHAP algorithms were employed for visual interpretation. Furthermore, time‐AUC and decision curve analysis demonstrated that the Aorsf model provided superior clinical benefits compared to other models.

**Conclusion:**

The PNI‐based Aorsf model effectively predicts long‐term survival outcomes after ablation therapy, making a significant contribution to HCC research by improving surveillance, prevention, and treatment strategies.

## 1 Introduction

Hepatocellular carcinoma (HCC) ranks as the fifth most commonly prevalent cancer globally [1]. The current Guidelines for the Diagnosis and Treatment of Primary HCC recommended locoregional ablation as one of the first‐line therapeutic approaches for HCC patients within Milan criteria (single nodule ≤ 5 cm or no more than three nodules ≤ 3 cm, without vascular invasion or extrahepatic metastasis) [2]. Nevertheless, the considerable rate of tumor relapse (> 60%) presents a substantial challenge in improving survival rates following ablation [3, 4, 5]. Several staging systems have been utilized to assess the risk and predict the prognosis in HCC, such as Barcelona Clinic Liver Cancer (BCLC) stage [6], American Joint Committee on Cancer tumor‐node‐metastasis (AJCC‐TNM) staging system [7] and Cancer of the Liver Italian Program (CLIP) score [8]. However, these conventional scoring systems have limitations as they mainly focus on tumor‐related factors and liver function reserve while disregarding gender, age, immune inflammatory indicators, nutritional indicators, and other nonanatomical factors. Numerous studies have consistently demonstrated the significant impact of systemic inflammation and nutritional status on HCC progression [9, 10, 11, 12, 13], as well as explored their correlations with clinical outcomes. However, for patients with early‐stage HCC who typically exhibit superior liver function reserve, smaller tumor burden, and minimal weight fluctuations during treatment, their nutritional status is often categorized as low‐risk or no‐risk. This classification restricts the effectiveness of commonly employed malnutrition screening tools such as the NRS‐2002 or royal‐free hospital nutritional prioritization tool [14]. Therefore, there is an urgent requirement for easily accessible alternative biomarkers that are effective in enhancing prognostic assessment accuracy while facilitating postoperative follow‐up and treatment categorization.

The prognostic nutritional index (PNI) has been proposed as a robust tool for assessing immune and nutritional aspects, making it a potentially powerful prognostic indicator in the intricate interplay between cancer and chronic liver inflammation in patients with HCC [15]. Recent studies by Song and Zhou have identified PNI as an important prognostic marker for HCC patients undergoing liver resection [16, 17]. Additionally, some studies have found that combining PNI with the GGT/ALT ratio can serve as a novel prognostic predictor for HCC patients within the Milan criteria after curative resection [18]. He et al. demonstrated that PNI and neutrophil‐to‐lymphocyte ratio (NLR) are independent predictors of survival in HCC patients after curative hepatectomy [19], leading to the development of a new nomogram based on PNI and NLR which provides personalized predictions for patient survival and recurrence. Furthermore, Jiang et al. highlighted PNI as a simple yet valuable predictor of overall survival (OS) in early‐stage HCC patients after ablation [20, 21]. However, these studies primarily focused on associating PNI with treatment prognosis in HCC rather than exploring its potential for developing machine learning (ML) prediction models to provide more comprehensive clinical outcomes.

To address these limitations, we have developed a series of ML models that integrate tumor burden data, liver function, PNI, and other blood laboratory test results to accurately predict the long‐term prognosis of patients undergoing local ablation for primary HCC. ML algorithms enable automated analytical model building by learning from clinical data, identifying patterns, and making decisions with minimal human intervention. This approach allows us to calculate the probability of future outcomes for individual patients and identify the most robust prediction model. To our knowledge, there is currently a dearth of research on utilizing ML models based on nutrition‐immune indicators to predict long‐term prognosis following primary HCC ablation. The integration of big data will enhance the accuracy of OS prediction models for HCC, improve the practicality of PNI in clinical settings, and provide valuable guidance for selecting appropriate candidates for local ablation procedures.

## 2 Materials and Methods

### 2.1 Study Design and Patient Data

**FIGURE 1:**
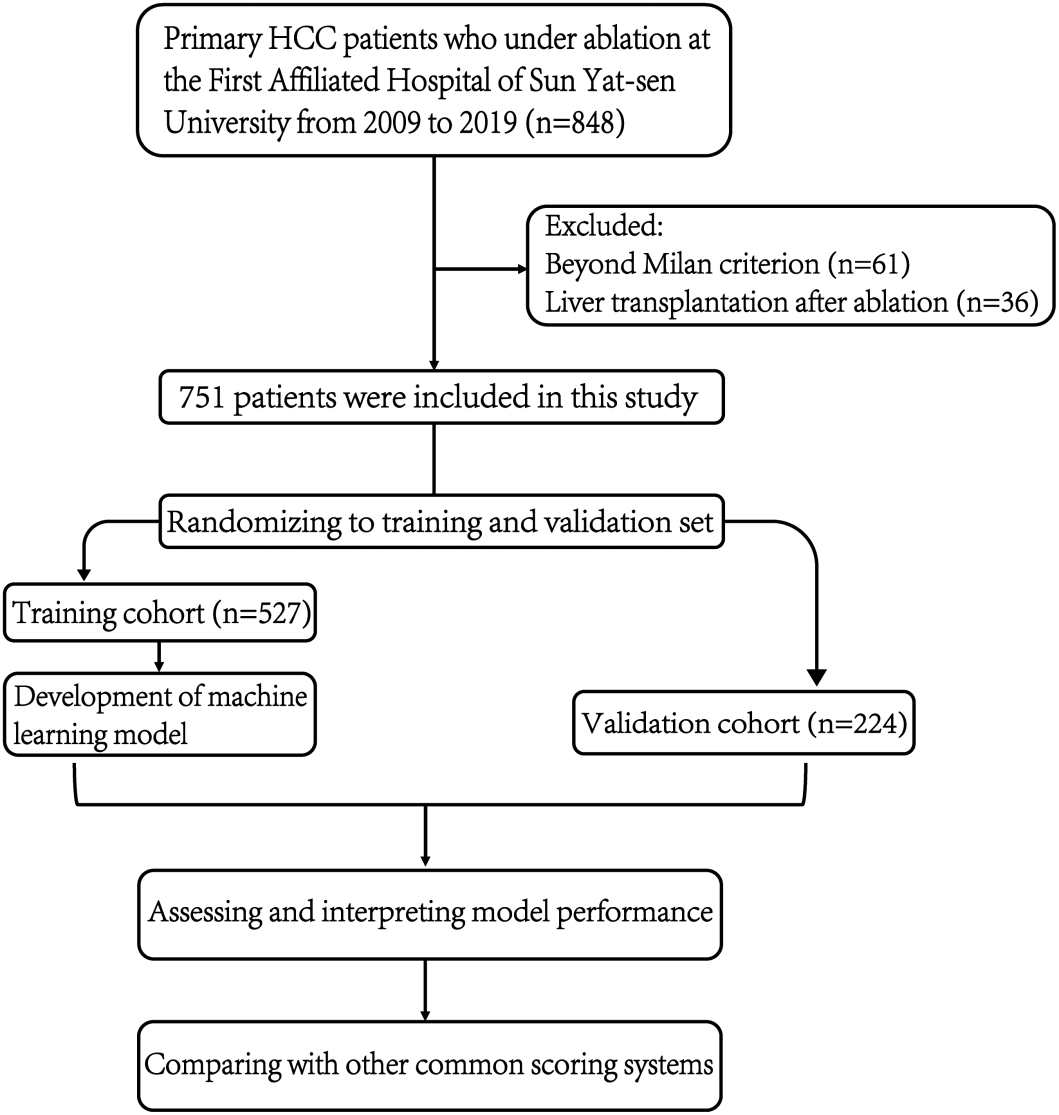
A flowchart is presented to illustrate the participant selection process employed in a clinical study. Initially, a total of 848 patients who had undergone local ablation for primary liver cancer were considered. Subsequently, 751 patients were retained after excluding individuals beyond the Milan criterion and those who underwent liver transplantation after ablation. The selection of these patients was based on their distinct characteristics, followed by the development of machine learning models and comparisons with alternative scoring systems. HCC, hepatocellular carcinoma.

The retrospective nature of this study allowed for the waiver of written informed consent, which was granted by the Institutional Ethics Committee of the First Affiliated Hospital of Sun Yat‐sen University, after obtaining approval from the ethics committee. Clinical data from 848 primary HCC patients who underwent local ablation at a large‐volume tertiary center in China (First Affiliated Hospital of Sun Yat‐sen University) from January 2009 to December 2019 were analyzed in accordance with guidelines provided by the European Association for the Study of the Liver (EASL) and the American Association for the Study of Liver Diseases (AASLD) [22, 23] to diagnose HCC. The inclusion criteria encompassed complete ablation in HCC patients, with a single tumor diameter ≤ 5 cm, or a tumor number ≤ 3 with a maximum diameter ≤ 3 cm, Child‐Pugh grade A or selected B liver function, and absence of vascular invasion, lymph node invasion, and extrahepatic metastasis. This study excluded 50 patients who had received anti‐cancer treatment prior to local ablation, 11 patients with a history of other malignant tumors, and 36 participants who underwent liver transplantation and were lost to follow‐up for more than 6 months. Ultimately, medical records were screened to identify data from 751 HCC patients. Subsequently, 527 patients were randomly assigned to the training set while 224 were assigned to the validation set based on a ratio of 7:3. Figure 1 illustrates the patient enrollment pathway and flowchart for building ML models based on clinical data.

### 2.2 Variable Collection and Feature Selection

The variable data with a missing ratio greater than 20% were excluded from our study. A total of 23 variables were collected for the development of predictive models. Some of the variables are explained as follows: PNI = (10 × Alb g/dL) + (0.005 × total lymphocyte count/mm^3^) [24]. APRI = (AST/ upper limit of normal × 100)/ platelet count (× 10^9^/L) [25]. Tumor number and size were evaluated using computerized tomography (CT) or magnetic resonance imaging (MRI) prior to ablation. Tumor size was defined as the diameter of the largest tumor. According to previous literature studies, AFP ≥ 200 μg/mL was used as the cut‐off value for AFP, tumor number > 1 as the cut‐off value for tumor number, and tumor size > 3 cm as the cut‐off value for tumor size. The multidisciplinary board of our hospital deliberated on diagnosis and treatment decisions. The ablation procedure and device have been previously described in detail in supplementary information. Patient follow‐up occurred at intervals of 1, 3, 6, 9, and12 months during the first year after ablation, followed by subsequent evaluations every 6 months until either tumor recurrence or mortality occurred. Every patient underwent routine liver function review, AFP analysis, and imaging studies including contrast‐enhanced ultrasound (CEUS), CT, and/or MRI during follow‐up. OS was operationally defined as the duration between HCC ablation date and death date or last follow‐up evaluation (January 2022).

Initially, univariate Cox regression analyses were performed to identify features significantly associated with OS. Subsequently, variables with a *p* < 0.05 in the univariate analysis were selected and utilized in the least absolute shrinkage and selection operator (LASSO) regression algorithm using the “glmnet” package [26, 27, 28]. The most significant features identified by LASSO from the training dataset were employed for multivariate Cox proportional hazards analyses, enabling the calculation of hazard ratios (HRs) along with their corresponding 95% confidence intervals (CIs), thereby identifying independent prognostic factors. All retained features obtained through this rigorous procedure were subsequently incorporated into the development of our predictive model.

### 2.3 Development and Validation of ML Predictive Models

In this study, we constructed a total of 19 ML models using the training cohort and subsequently compared them to determine the optimal prognostic model. All learners in this study utilized the random forest learning method, which was implemented using the R package “mlr3.” (Detailed codes and construction processes can be found at https://github.com/mlr‐org). The models underwent resampling via 10‐fold cross‐validation, with 80% of the samples being selected at each iteration. The “mlr3” package was utilized to employ benchmark functions for model discrimination. This enables us to objectively evaluate the performance of various algorithms, models, or methods on a specific task. Discrimination of the models was evaluated using the C‐index, and visualization of results was performed using the “autograph” function. The top 10 models were selected based on their performance. Among these 10 models, selection of the best prognostic model was determined by evaluating multiple criteria including the C‐index, concordance‐discordance area under curve (C/D AUC), and Brier score. We used “survex” package in R for survival models explanation and performance examination [29]. Then, we employ the “timeROC” package in R to assess the time‐dependent area under curve (AUC) for evaluating the performance of our time‐dependent survival model [30]. Subsequently, an assessment was conducted on the time‐dependent feature importance of survival models, followed by the generation of partial dependence plots (PDP) for each individual feature to elucidate their influence on survival. To interpret predictions made by our best model, we employed the SHapley Additive exPlanations (SHAP) framework. In this study, the prognostic performance of the model was compared with other scoring systems, including CLIP [8], BCLC [6], AJCC‐TNM [7], Japan Integrated Staging (JIS) [31], Chinese University Prognostic Index (CUPI) [32], French/GETCH [33], and Okuda scoring systems [34], using time‐AUC and decision curve analysis (DCA), which demonstrated the degree of consistency between the predicted risk and the actual risk of the model was evaluated. The validation set data were used to evaluate the predictive performance of the model using the above methods.

### 2.4 Statistical Analysis

**FIGURE 2:**
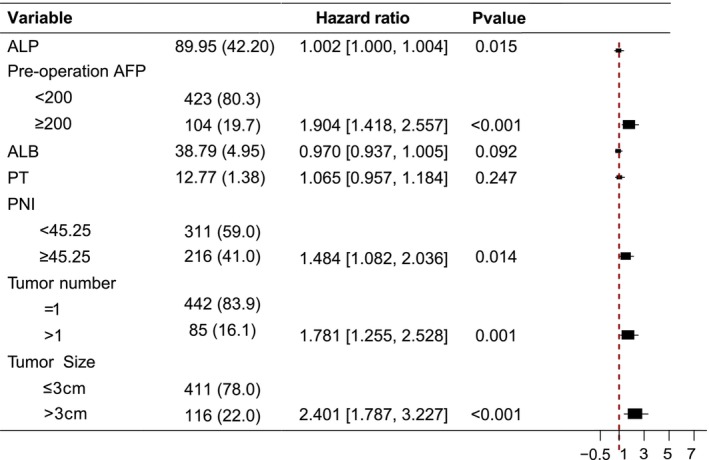
Forest plot summary of multivariate Cox analyses of prognosis. Multivariate analyses were carried out using the clinical covariates in the training cohort based on overall survival. AFP, alpha‐fetoprotein; ALB, albumin; ALP, alkaline phosphatase; PNI, prognostic nutritional index; PT, prothrombin time.

In all tables, continuous variables are reported as means and standard deviations, and compared using the Student's *t*‐test; while the risk ratio (HR) in Figure 2 and Figure S2 is presented as median and interquartile range (IQR), and compared using the Cox regression analysis. Categorical variables were presented as percentages and compared by the chi‐squared test. Survival curves were calculated with the Kaplan–Meier method and compared with the log‐rank test. Given that rapid assessment of OS with a cut‐off value may be useful in routine clinical practice, we stratified patients into low‐PNI and high‐PNI subgroups according to the optimal cut‐off threshold determined by the maximum selected log‐rank statistic. The statistical analysis of the data was conducted using R software (version 4.2.0.). A significance level of *p* < 0.05 was considered statistically significant.

## 3 Results

### 3.1 Baseline Characteristics of Patients

Over a 10‐year follow‐up period, a total of 751 patients were retrospectively included in the study. The baseline characteristics of the patients are shown in Table 1. There were no significant differences in baseline characteristics observed between the training and validation groups. Patients were stratified into low‐PNI and high‐PNI groups based on the PNI cut‐off value (Figure S1). The proportion of patients classified as high‐PNI was 59.01% (311 out of 527) and 65.18% (146 out of 224) for the training set and validation set, respectively. Across all datasets, age, ALP, APRI, AST, PT, GGT, and TBIL levels exhibited significantly lower values in the high PNI group compared to the low PNI group, and the differences were statistically significant. Conversely, ALB, LYM, NEU, PLT, and WBC showed significantly higher values in the high PNI group than in the low PNI group, and the differences were statistically significant (Table S1).

**TABLE 1:** Baseline characteristics of HCC patients in different cohorts.

	Train	Valid	Overall	
	(*n* = 527)	(*n* = 224)	(*n* = 751)	*p*
Age	56.9 (11.7)	56.7 (11.4)	56.8 (11.6)	0.969
ALB	38.8 (4.95)	38.8 (4.83)	38.8 (4.91)	0.996
ALP	89.9 (42.2)	86.9 (35.1)	89.0 (40.2)	0.586
ALT	39.3 (29.5)	37.8 (27.6)	38.9 (28.9)	0.784
APRI	0.375 (0.359)	0.369 (0.334)	0.373 (0.351)	0.975
AST	40.4 (28.3)	39.5 (26.0)	40.1 (27.6)	0.901
GGT	74.1 (87.0)	77.0 (94.4)	74.9 (89.2)	0.926
LYM	1.77 (0.644)	1.67 (0.631)	1.74 (0.641)	0.139
NEU	3.02 (1.43)	3.07 (1.64)	3.04 (1.50)	0.945
PLT	145 (71.2)	142 (71.2)	144 (71.2)	0.886
PNI	46.7 (6.74)	46.3 (6.49)	46.6 (6.66)	0.679
PT	12.8 (1.38)	12.8 (1.29)	12.8 (1.35)	0.862
TBIL	17.6 (9.28)	16.8 (9.06)	17.4 (9.22)	0.473
WBC	5.53 (1.80)	5.40 (1.93)	5.49 (1.84)	0.671
Child‐pugh				
Stage A	505 (95.8%)	209 (93.3%)	714 (95.1%)	0.344
Stage B	22 (4.2%)	15 (6.7%)	37 (4.9%)	
HBsAg				
−	79 (15.0%)	31 (13.8%)	110 (14.6%)	0.92
+	448 (85.0%)	193 (86.2%)	641 (85.4%)	
HBVDNA				
< 100 × 10^2^	350 (66.4%)	142 (63.4%)	492 (65.5%)	0.728
≥ 100 × 10^2^	177 (33.6%)	82 (36.6%)	259 (34.5%)	
HCVAb				
−	497 (94.3%)	218 (97.3%)	715 (95.2%)	0.209
+	30 (5.7%)	6 (2.7%)	36 (4.8%)	
Preoperation AFP				
< 200	423 (80.3%)	183 (81.7%)	606 (80.7%)	0.902
≥ 200	104 (19.7%)	41 (18.3%)	145 (19.3%)	
PS_score				
0	525 (99.6%)	220 (98.2%)	745 (99.2%)	0.141
1	2 (0.4%)	4 (1.8%)	6 (0.8%)	
Sex				
Male	448 (85.0%)	191 (85.3%)	639 (85.1%)	0.996
Female	79 (15.0%)	33 (14.7%)	112 (14.9%)	
Tumor Number				
1	442 (83.9%)	195 (87.1%)	637 (84.8%)	0.539
> 1	85 (16.1%)	29 (12.9%)	114 (15.2%)	
Tumor Size				
≤ 3	411 (78.0%)	170 (75.9%)	581 (77.4%)	0.821
> 3	116 (22.0%)	54 (24.1%)	170 (22.6%)	
BCLC				
0	156 (29.6%)	73 (32.6%)	229 (30.5%)	0.718
A	371 (70.4%)	151 (67.4%)	522 (69.5%)	
CLIP				
0	371 (70.4%)	155 (69.2%)	526 (70.0%)	0.802
1	136 (25.8%)	64 (28.6%)	200 (26.6%)	
2	20 (3.8%)	5 (2.2%)	25 (3.3%)	
TNM				
T1a	156 (29.6%)	73 (32.6%)	229 (30.5%)	0.825
T1b	286 (54.3%)	122 (54.5%)	408 (54.3%)	
T2	85 (16.1%)	29 (12.9%)	114 (15.2%)	
French				
0	442 (83.9%)	195 (87.1%)	637 (84.8%)	0.539
1	85 (16.1%)	29 (12.9%)	114 (15.2%)	
CUPI				
Low risk	513 (97.3%)	219 (97.8%)	732 (97.5%)	0.944
Intermediate risk	14 (2.7%)	5 (2.2%)	19 (2.5%)	
JIS				
0	442 (83.9%)	195 (87.1%)	637 (84.8%)	0.539
1	85 (16.1%)	29 (12.9%)	114 (15.2%)	
Okuda				
Stage I	442 (83.9%)	195 (87.1%)	637 (84.8%)	0.539
Stage II	85 (16.1%)	29 (12.9%)	114 (15.2%)	

Abbreviations: AFP, alpha fetoprotein; AJCC‐TNM, American Joint Committee on Cancer tumor‐node‐metastasis staging system; ALB, albumin; ALBI, albumin‐bilirubin index; ALP, Alkaline phosphatase; ALT, alanine aminotransferase; APRI, aspartate aminotransferase‐to‐platelet ratio index; AST, aspartate aminotransferase; BCLC, Barcelona Clinic Liver Cancer; CLIP, Cancer of the Liver Italian Program score; CUPI, Chinese University Prognostic Index; GGT, gamma‐glutamyl transferase; HBsAg, hepatitis B surface antigen; HBV‐DNA, hepatitis B virus DNA; HCVAb, hepatitis C virus antibody; JIS, Japan Integrated Staging; LYM, lymphocyte; NEU, neutrophil; PLT, platelet count; PNI, prognostic nutritional index; PT, prothrombin time; TBIL, total bilirubin; WBC, white blood cell count.

### 3.2 Variables Selected

The factors influencing OS in HCC were further investigated through a univariate analysis. This analysis revealed significant associations between various factors, including age, ALP, LYM, preoperation AFP, AST, GGT, TBIL, ALB, PLT, PT, PNI, APRI, Child‐pugh stage, tumor number, and tumor size (Figure S2). To enhance the generalization ability of the model by reducing coefficients of certain variables and filtering out less relevant characteristics, we employed LASSO regression which is commonly used for data dimension reduction. Following LASSO regression screening, ALP, preoperation AFP, ALB, PT, PNI, tumor size, and tumor number were identified as closely related to the OS of HCC patients after ablation (Figure S3). These seven selected features were used in the multivariate Cox regression analysis which identified several independent prognostic factors for OS in HCC patients after local ablation: ALP (HR: 1.002, 95% CI: 1.001–1.004, *p* = 0.015), preoperation AFP levels (HR: 1.904, 95% CI: 1.418–2.557, *p* = < 0.001), PNI (HR: 1.484, 95% CI: 1.082–2.036, *p* = 0.014), tumor number (HR: 1.781, 95% CI: 1.255–2.528, *p* = 0.001), tumor size (HR: 2.401, 95% CI: 1.787–3.227, *p* < 0.001) (Figure 2). We input these five factors into the ML algorithms to develop and validate the predictive model for OS after local ablation.

### 3.3 Construction of Different ML Model in Predicting Survival Probability

**FIGURE 3:**
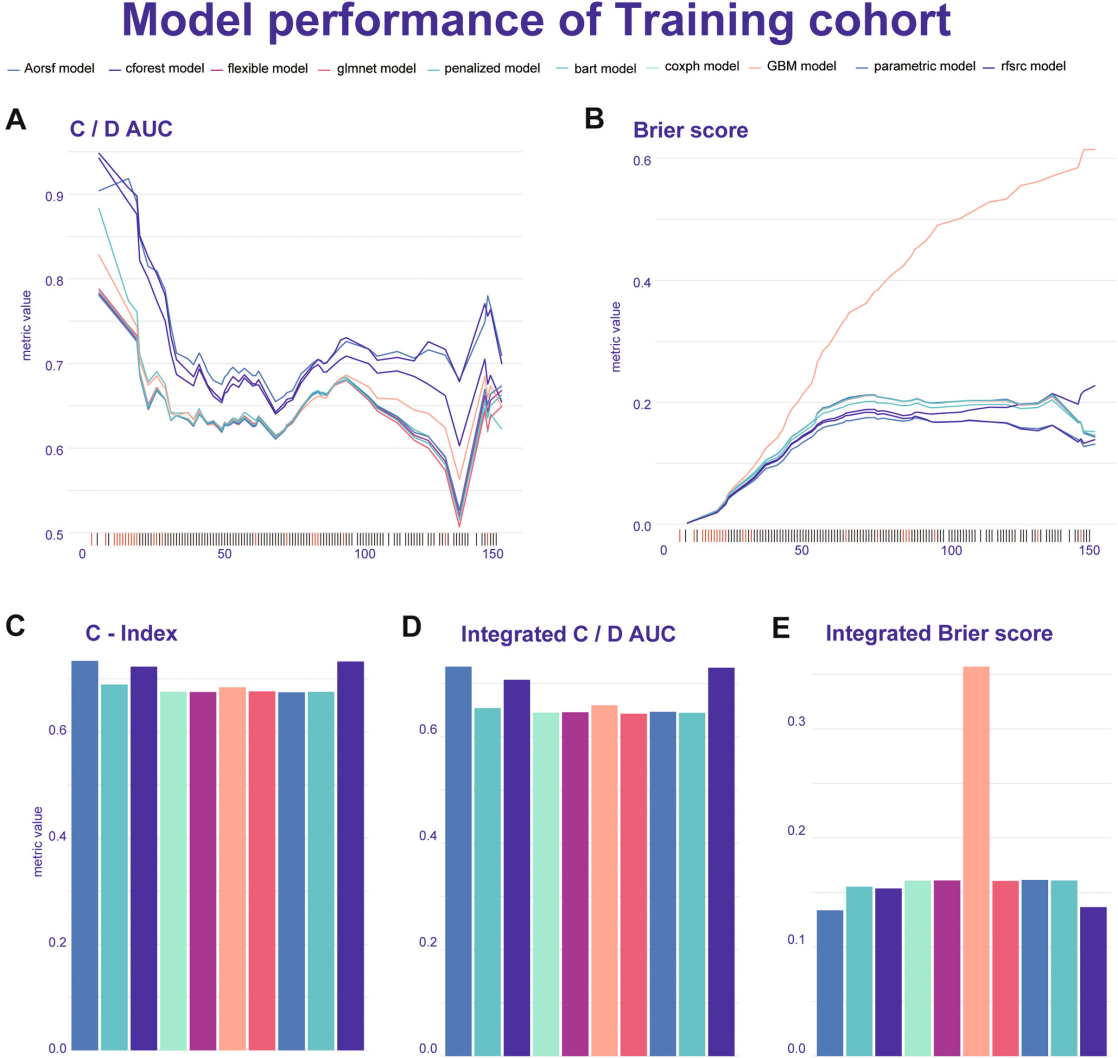
The performance evaluation of the top 10 machine learning models is assessed using metrics such as C/D AUC and Brier score, presented through a time‐dependent line graph in training cohort. The bar graph shows the integrated metrics of the model's overall performance. C‐index, Concordance Index; C/D AUC, concordance‐discordance area under curve.

**FIGURE 4:**
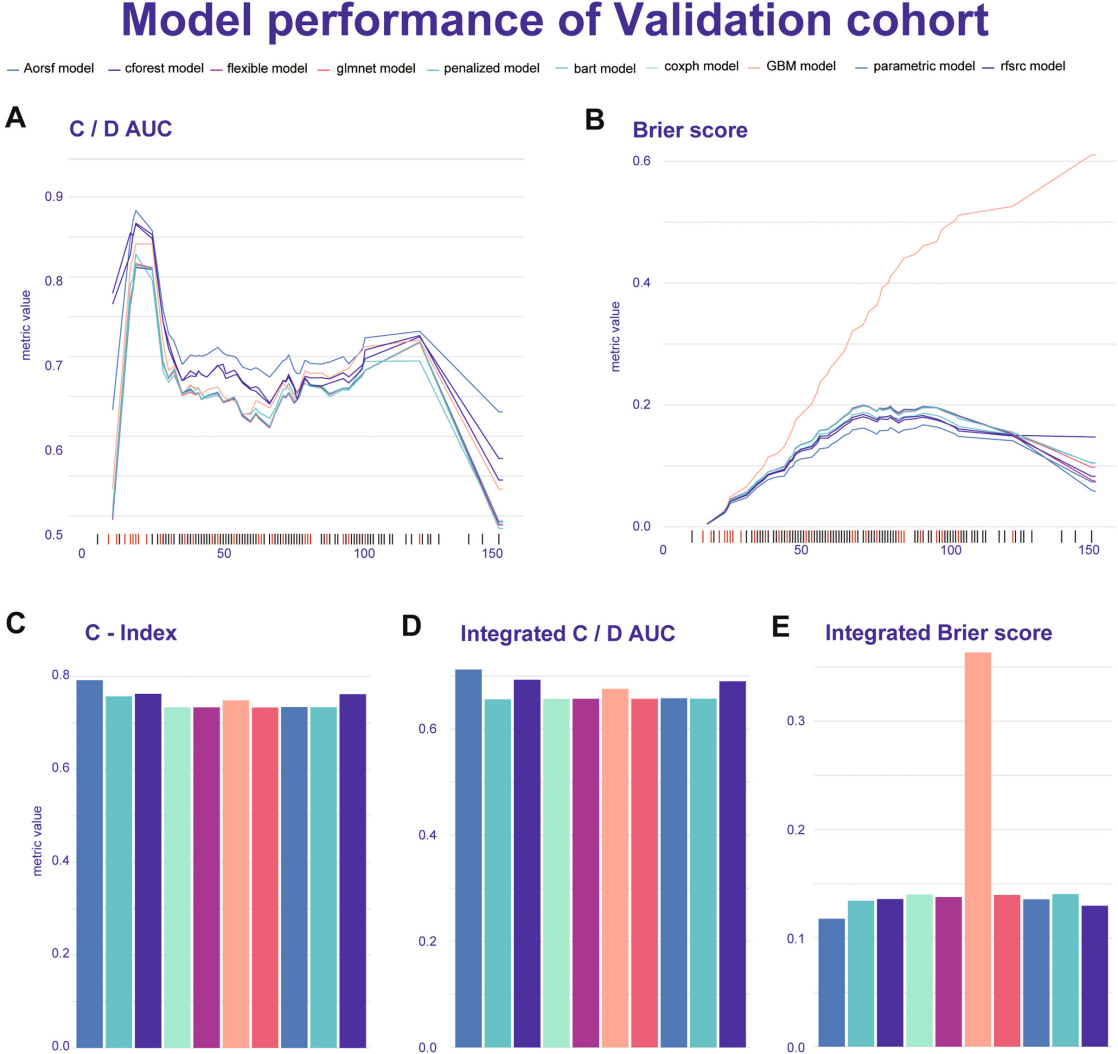
The performance evaluation of the top 10 machine learning models is assessed using metrics such as C/D AUC and Brier score, presented through a time‐dependent line graph in validation cohort. The bar graph shows the integrated metrics of the model's overall performance. C‐index, Concordance Index; C/D AUC, concordance‐discordance area under curve.

**FIGURE 5:**
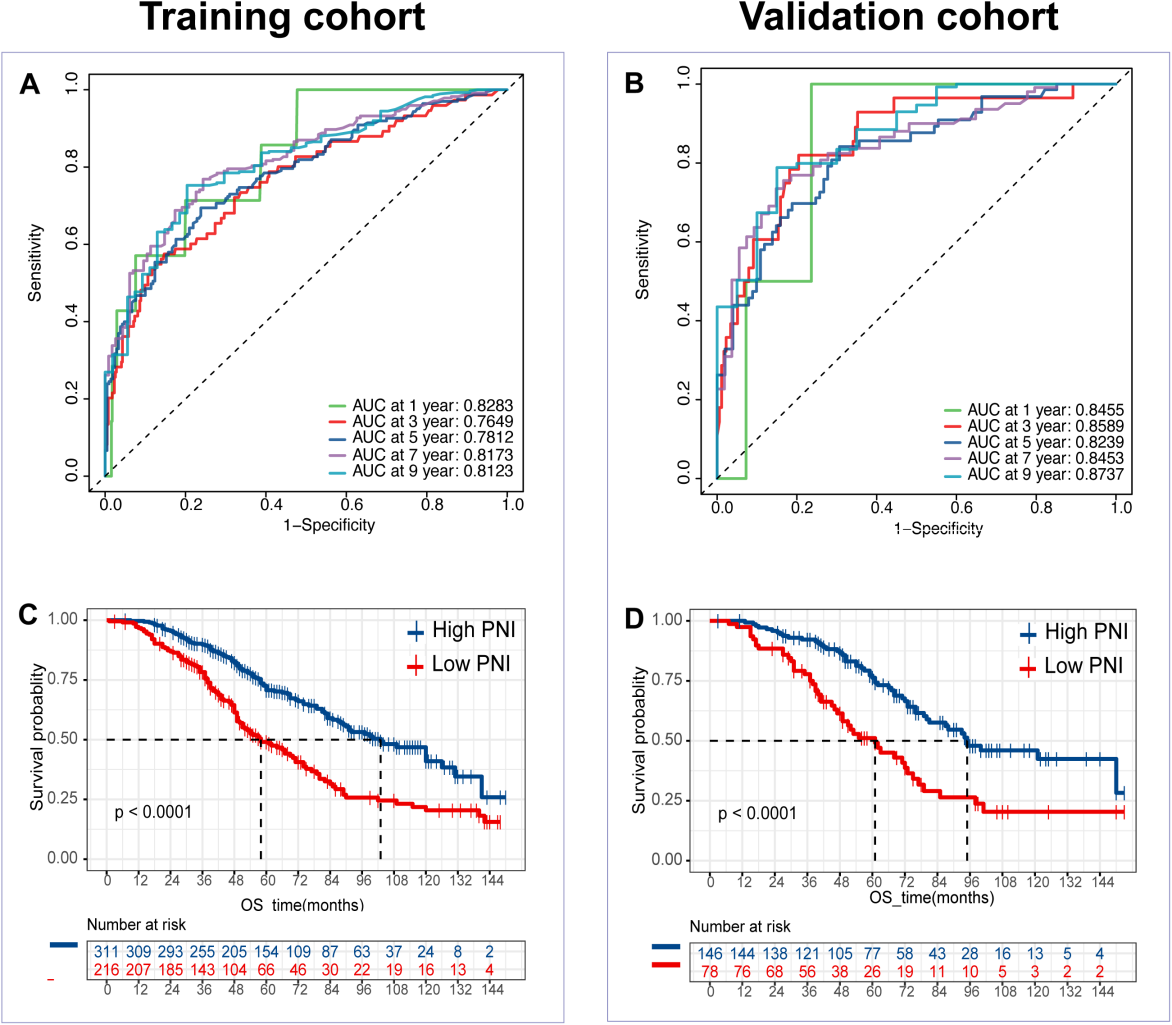
The performance of the Aorsf model in predicting the prognosis of local ablation of primary HCC is illustrated in the charts. (A) The time‐dependent area under the curves (AUCs) for overall survival at 1, 3, 5, 7, and 9 years were found to be 0.828, 0.765, 0.781, 0.817 and 0.812, respectively, in the training set. (B) In the validation set, corresponding AUCs were found to be as follows: At year one – 0.846; at year three – 0.859; at year five – 0.824; at year seven – 0.845; and at year nine – 0.874. Graphs Show comparison Kaplan–Meier curves of (C) OS between high‐PNI and low‐PNI patients in training cohort. (D) OS between high‐PNI and low‐PNI patients in validation cohort. HCC, hepatocellular carcinoma; OS: overall survival; PNI, prognostic nutritional index.

The performance of the 19 ML models was evaluated based on the average C‐index, leading to the selection of the top 10 algorithms for further comparison (Figure S4). Among the 10 prediction models, the Aorsf model exhibited superior predictive ability, demonstrating C/D AUC ranging from 0.79 to 0.918 for the training set, while achieving C/D AUC ranging from 0.628 to 0.880 for the validation set (Figures 3A and 4A). The C‐index value of 0.736 and integrated C/D AUC value of 0.733 for the training set, while C‐index value of 0.793 and integrated C/D AUC value of 0.713 for the validation set (Figures 3C,D and 4C,D). Additionally, compared to other models, Aorsf displayed better model performance and fitness as evidenced by Brier scores ranging from 0.002 to 0.174 for the training set, while achieving Brier scores ranging from 0.004 to 0.167 for the validation set (Figures 3B and 4B). The integrated Brier scores value of 0.133 for the training set and 0.117 for the validation set (Figures 3E and 4E). Considering the 1‐, 3‐, 5‐, 7‐, and 9‐year OS aspects, the time‐dependent area under the curves (AUCs) of the training set were found to be 0.828, 0.765, 0.781, 0.817, and 0.812, respectively (Figure 5A). Similarly, for the validation set, the AUCs were observed to be 0.846, 0.859, 0.824, 0.845, and 0.874 for corresponding time points (Figure 5B). The survival curve demonstrated a significantly lower mortality rate in the high PNI group compared to the low PNI group, indicating substantial disparities between the two prognostic models in both the training and validation sets (Figure 5C,D). These results highlight the remarkable predictive efficacy of our Aorsf model.

Subsequently, we further explored the robustness of our ML predictive model through subgroup analysis by stratifying patients based on preoperation AFP levels, tumor size, and tumor number (Figure S5). In the preoperation AFP < 200 subgroups, the high‐PNI group exhibited a significantly longer median OS compared to the low‐PNI group in the training set (120 vs. 66 months; *p* < 0.0001) (Figure S5A). In the validation cohorts, there were notable differences in median OS between the high‐ and low‐PNI groups: 100 versus 71 months (*p* < 0.00026) (Figure S5D). Similar findings were observed when comparing OS rates between high‐ and low‐PNI groups of patients within subgroups based on tumor size ≤ 3 cm (Figure S5B,E). Subgroup analysis of patients with tumor number = 1 yielded consistent results (Figure S5C,F). The Aorsf model consistently exhibits reliable discriminative ability in all various subgroups for both the training and validation sets, highlighting its exceptional stability and accuracy.

### 3.4 Interpretation of the Aorsf Model

**FIGURE 6:**
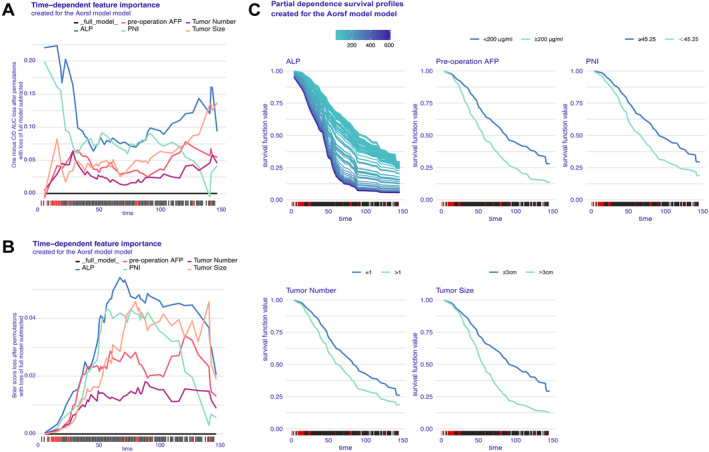
Partial dependence survival profiles of independent factors influencing overall survival in the Aorsf model, specifically examining the survival distribution curve of characteristic variables for determining final survival outcomes. AFP, alpha‐fetoprotein; ALP, alkaline phosphatase; PNI, prognostic nutritional index.

To evaluate the significance of the ranked variables, we utilize the variable importance graph, where their descending order indicates their utmost importance. It is important to note that the loss function exhibits time‐dependency. The ALP variables hold the highest rank in inducing a maximum increase in the loss function, closely followed by PNI (Figure 6A). These variables play a crucial role in predicting outcomes within our model. Interestingly, these findings align with those obtained from evaluating Brier score loss, confirming both the robustness of our model and the criticality of these variables for accurate predictions (Figure 6B). Furthermore, we conducted a comprehensive evaluation and global interpretation of the time‐dependent feature importance in the survival model and extended the PDP for each feature to evaluate their impact on survival. The bands representing the continuous variable ALP and the categorical variables PNI, preoperative AFP, tumor size, and tumor number were significantly distant from each other, indicating that even slight variations in their values could result in substantial disparities in the predicted survival function. The results depicted in Figure 6C demonstrate that preoperation AFP levels < 200, PNI values ≥ 45.25, tumor sizes ≤ 3 cm, tumor number = 1, and increased ALP levels all exerted a favorable influence on OS. This demonstrates the significance of incorporating PNI as a prognostic indicator alongside traditional tumor markers, liver function, and tumor burden. SHAP values offer insights into the predictive characteristics of each patient and the contribution of each characteristic to the prediction of OS. Each curve represents a specific characteristic, with time on the x‐axis and the position of the baseline on the y‐axis indicating positive or negative correlation. The individual strength plots for Patient 1 and Patient 2 are depicted in Figure S6, respectively. For instance, in Patient 1, an increase in tumor number enhances individual's odds of survival while other variables decrease them (Figure S6A,B). In contrast, for Patient 2, both tumor number and tumor size elevate survival odds whereas the remaining three variables diminish them (Figure S6C,D).

### 3.5 Assessing and Comparing Model Performance

**FIGURE 7:**
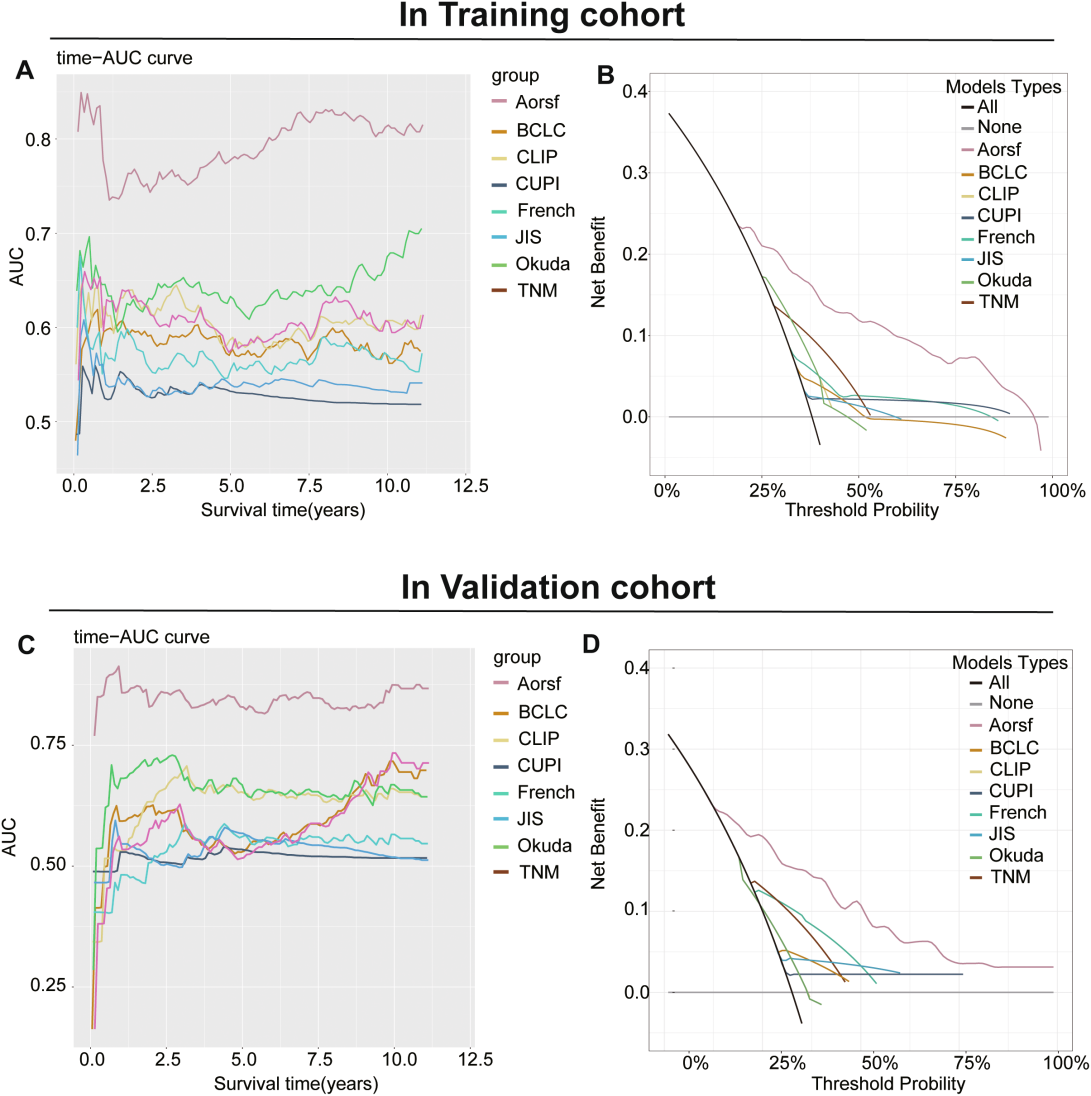
(A, B). Predict survival prognosis in the training set by comparing the time‐AUC curve and decision curve analysis between the Aorsf model and other scoring systems. (C, D). Predict survival prognosis in the validation set by comparing the time‐AUC curve and decision curve analysis between the Aorsf model and other scoring systems. Aorsf, Oblique random survival forests; AJCC TNM, American Joint Committee on Cancer tumor‐node‐metastasis; BCLC, Barcelona Clinic Liver Cancer; CLIP, Cancer of the Liver Italian Program; CUPI, Chinese University Prognostic Index; JIS, Japan Integrated Staging Score.

The predictive accuracy of the Aorsf model was verified using several other methods. Time‐dependent AUC curve analysis was employed to evaluate the prognostic performance of different staging systems. In the training cohort, the Aorsf model exhibited an area under the time‐dependent curve ranging from 0.735 to 0.829 over a follow‐up period of 10 to 120 months. Similarly, in the validation set cohort, the Aorsf model demonstrated an area under the time‐dependent curve ranging from 0.765 to 0.913 across the same time range (Figure 7A,C). These findings highlight that the diagnostic ability of the Aorsf model is superior and its corresponding AUC outperforms international staging systems including CLIP, AJCC‐TNM, JIS, CUPI, French, and Okuda models. Additionally, decision curve analysis (DCA) was performed to compare the performance of the Aorsf model with other widely used scoring systems evaluated in this study (Figure 7B,D). The Aorsf model demonstrated superior positive net benefits compared with all other systems, which was demonstrated in both the training and validation sets.

## 4 Discussion

In this study, we developed and validated ML models using clinical data reflecting inflammatory and nutritional factors to predict individual OS outcomes after ablation in a long‐term follow‐up cohort of patients with early‐stage HCC. The Aorsf model demonstrates superior performance and discrimination compared to the other 18 predictive ML models and common scoring systems. Through a comprehensive analysis of the predictive model, we have effectively identified the key risk factors influencing survival analysis in patients following HCC ablation. This elucidation not only enhances clinicians' comprehension of the underlying principles but also facilitates informed utilization of prognostic outcomes, thereby optimizing patient treatment strategies.

The existing BCLC, AJCC‐TNM staging systems have been gradually applied in clinical practice for prognostic risk assessment, but they only involve tumor size, number, vascular invasion, and distant metastasis, while ignoring the prognostic impact of systemic nutrition and immune status. In recent years, nutritional status has also been identified as a major determinant of inflammatory status, immune function, and therapeutic response. Although malnutrition is a prevalent complication in patients with cirrhosis or liver malignancies, it often goes undiagnosed and is not included in risk assessment [35, 36]. The PNI, which comprises albumin levels and lymphocytes, may serve as an easily available parameter to describe the link between nutritional and immune status. As a component of PNI, serum albumin is produced by liver parenchyma and reflects the reserve function of the liver, with important physiological functions such as antioxidant, endothelial stabilization, and immune regulation [37]. Lymphocytes, on the other hand, play an important role in tumor immune monitoring [38]. Increasing evidence supports that the interaction between infiltrating immune cells and tumor components is an important driver of tumor progression and therapeutic sensitivity [39]. Therefore, in HCC, a tumor associated with inflammation, PNI is particularly important [40, 41, 42].

Previous studies have found that PNI is an independent factor for evaluating the prognosis of HCC resection [43, 44]. With the development of local ablation therapy, retrospective studies of patients with early HCC who received RFA found that preoperative PNI levels were independent prognostic factors for OS and RFS in multivariate analysis. Saitsu's team confirmed that preoperative PNI levels are simple and novel predictors of survival and recurrence in patients with early HCC who received RFA [45]. Liang et al. confirmed that among HCC patients receiving ultrasound‐guided percutaneous RFA, the OS of patients with preoperative PNI > 45 was better than that of patients with PNI < 45. In the multivariate Cox proportional hazards model, preoperative PNI was considered as an independent prognostic factor for OS, and there was a nearly linear relationship between PNI and OS [46]. Jiang et al. have demonstrated that PNI is a straightforward and effective predictor of OS following local ablation in patients with early‐stage HCC. Therefore, we focus on the PNI indicator and combine it with other clinically relevant indicators to predict the prognosis of primary HCC.

We use ML methods to deeply mine and analyze multidimensional medical data to better apply it to prognosis prediction. To find the optimal ML model, we used the “tune_nested” function in the “mlr3” package to optimize hyperparameters and select the parameters with the highest C‐index value for further comparison. To ensure fairness and accuracy, we used the “benchmark” function in “mlr3” to compare the models. The final selected “Aorsf” model showed better precision and stability in three different indicators (C‐index, comprehensive C/D AUC, and comprehensive Brier score). We conducted a global explanation. We used the model_parts() function to examine the specific impact of each variable on the model's predictive performance, considering the time dynamics. Throughout the entire follow‐up period, we used both the Brier score and the C/D AUC loss function to evaluate the accuracy of the predictions, explicitly incorporating time into the analysis, meaning that the effect of each variable may differ at different points in time. Next, we used the model_profile() function to PDP curves, which provide a visual representation of the average impact of the model's predictions when a particular variable takes on different values. Additionally, we utilized Survshap (an innovative algorithm that is model‐agnostic) to provide local explanations for survival models that predict individualized survival curves. SHAP calculates the contribution of each feature to the prediction outcome for a specific patient, thus quantifying the impact of each feature on the model's prediction. Therefore, the Aorsf model we obtained not only demonstrated excellent predictive ability but also had good interpretability. This played a crucial and positive role in evaluating patients' long‐term prognosis and promptly formulating treatment strategies. To our knowledge, this is the first study constructing a ML model using PNI for assessing survival analysis in relation to ablation of primary HCC.

There are several limitations to our study. First, the patient data utilized in this research were obtained retrospectively from a solitary institution, and no external validation was conducted. Second, this study was carried out exclusively in China, where the majority of HCC patients have a history of HBV infection. However, previous studies have indicated that etiological factors and liver background have had minimal impact on survival prognosis [47, 48, 49]. During the follow‐up period, we were unable to investigate the impact of HCC recurrence or other HCC treatments on the model performance. Lastly, it is important to note that this model has not been implemented in clinical practice. To validate the model's effective generalizability, prospective, and external validation are needed.

## 5 Conclusions

We developed Aorsf model consisting of clinical variables reflecting inflammatory and nutritional factors, the model exhibited adequate performance and individualized predictive ability. Consequently, this visual Aorsf model may help physicians with decision‐making before ablation for HCC in clinical practice and trials.

## Author Contributions

**Nan Zhang:** data curation (equal), methodology (equal), supervision (equal), writing – original draft (equal). **Ke Lin:** formal analysis (equal), methodology (equal), software (equal), validation (equal). **Bin Qiao:** methodology (equal), supervision (equal), visualization (equal). **Liwei Yan:** investigation (equal), methodology (supporting), supervision (equal), visualization (supporting). **Dongdong Jin:** data curation (equal), methodology (supporting), resources (equal). **Daopeng Yang:** data curation (equal), formal analysis (supporting), validation (equal). **Yue Yang:** data curation (equal), validation (equal). **Xiaohua Xie:** project administration (equal), supervision (supporting). **Xiaoyan Xie:** project administration (equal), supervision (equal). **Bowen Zhuang:** conceptualization (equal), project administration (equal), supervision (equal), writing – review and editing (equal).

## Conflicts of Interest

The authors declare no conflicts of interest.

## Supporting information


Figure S1.

Figure S2.

Figure S3.

Figure S4.

Figure S5.

Figure S6.

Data S1.

Table S1.

## Data Availability

All analyzed data are included in this published article, and the original data used in the current study can be obtained from the corresponding author upon reasonable request.
