# Supplementary figures and images for: Machine Learning Model Based on Prognostic Nutritional Index for Predicting Long‐Term Outcomes in Patients With HCC Undergoing Ablation
Source: Cancer Med. 2024 Oct 23;13(20):e70344. doi: 10.1002/cam4.70344 (PMC11496905; doi:10.1002/cam4.70344)

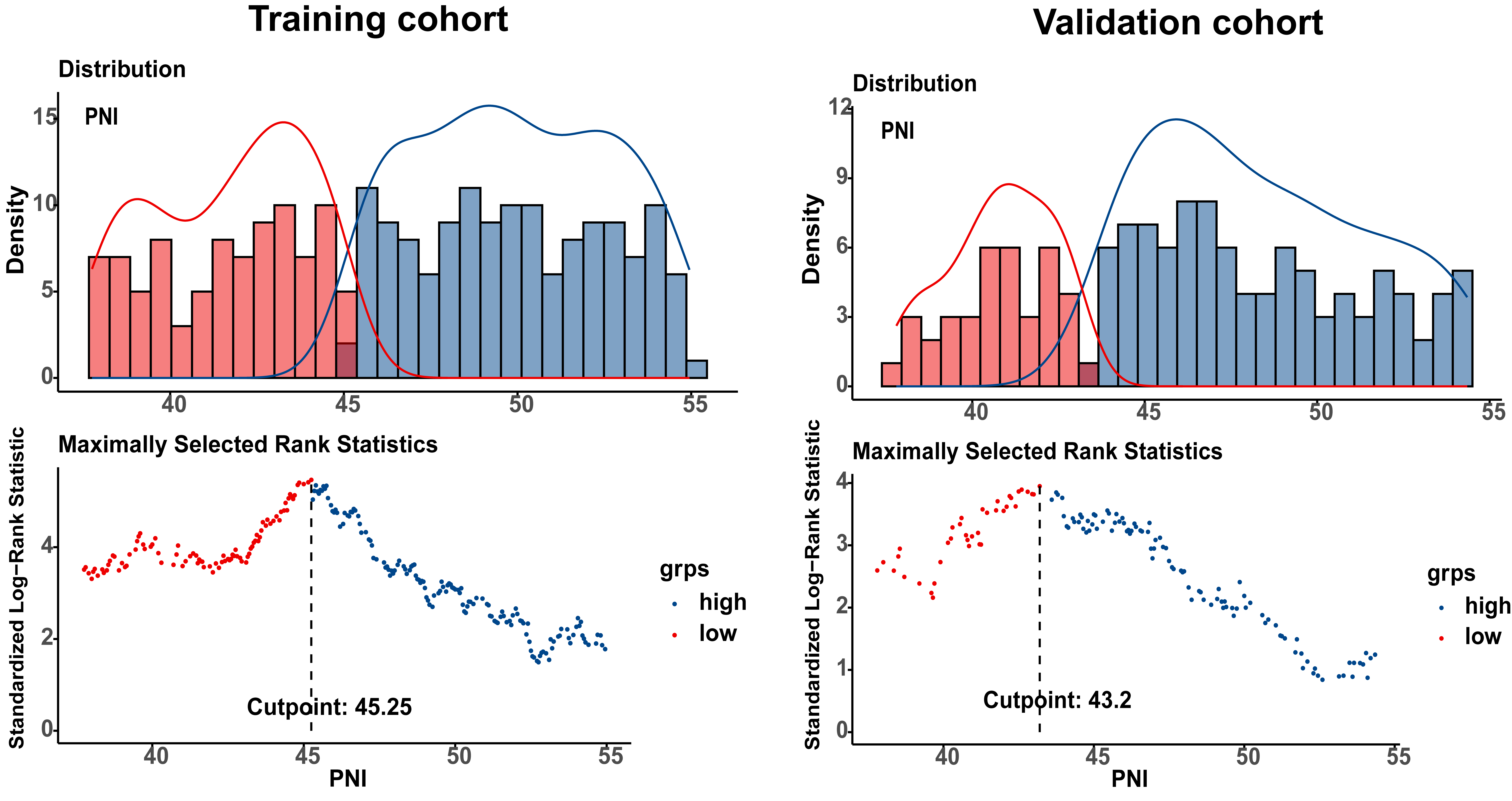

Supplement: Supplementary file 1 — Figure S1. [file CAM4-13-e70344-s008.tif]

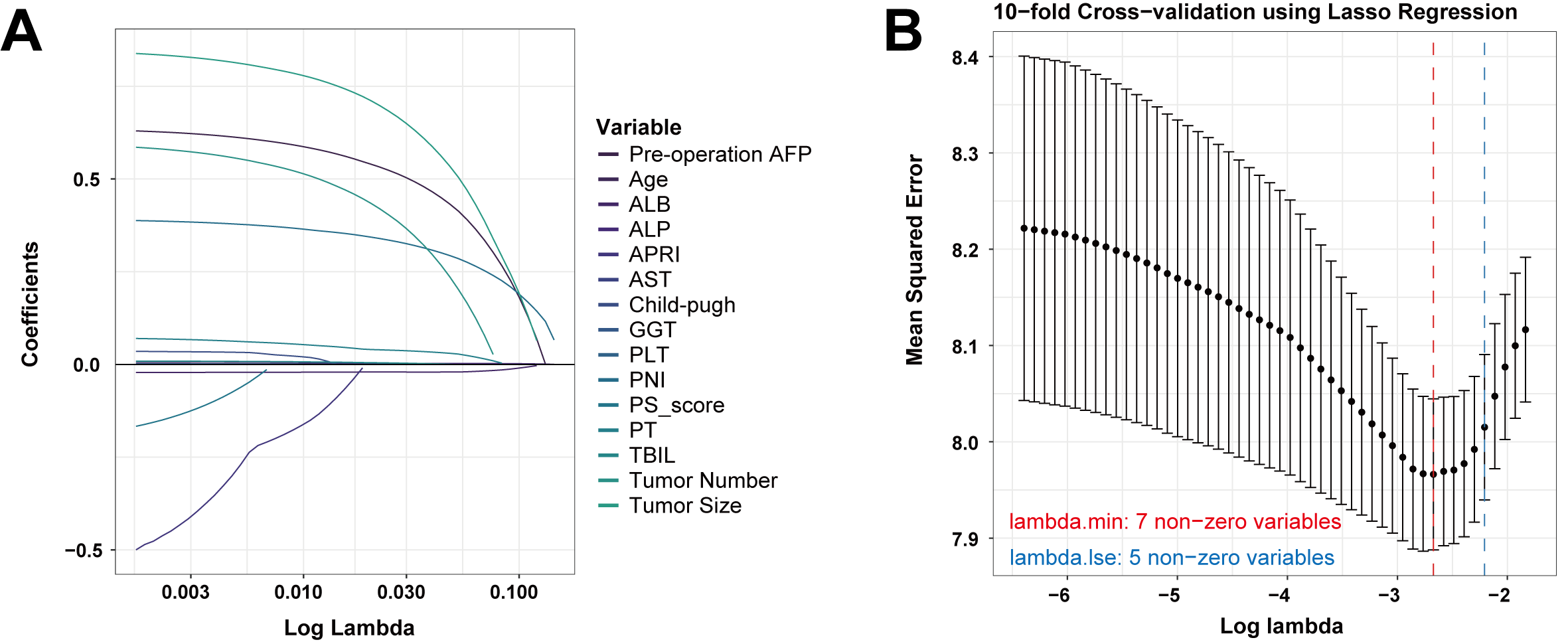

Supplement: Supplementary file 3 — Figure S3. [file CAM4-13-e70344-s007.tif]

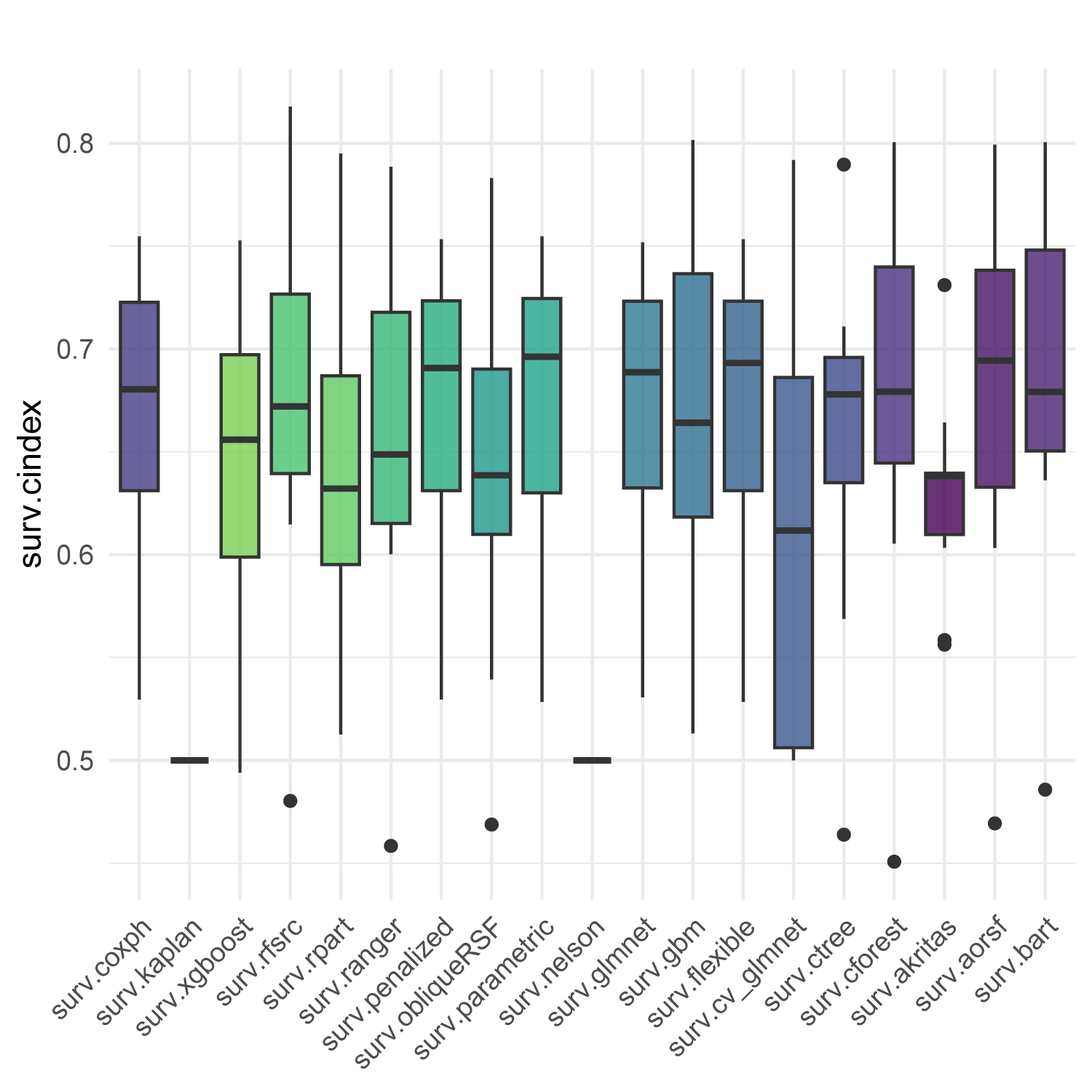

Supplement: Supplementary file 4 — Figure S4. [file CAM4-13-e70344-s006.tif]

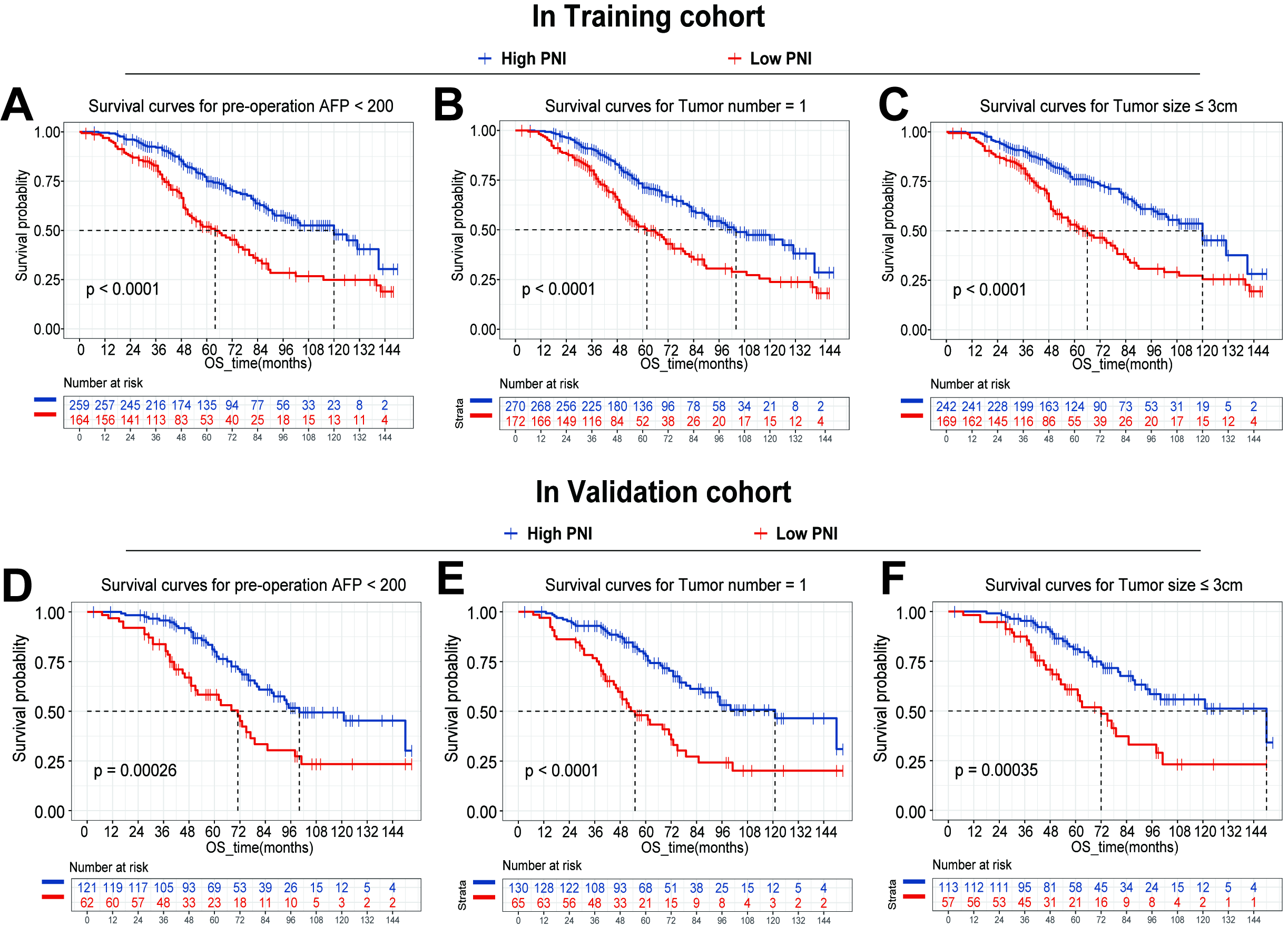

Supplement: Supplementary file 5 — Figure S5. [file CAM4-13-e70344-s003.tif]

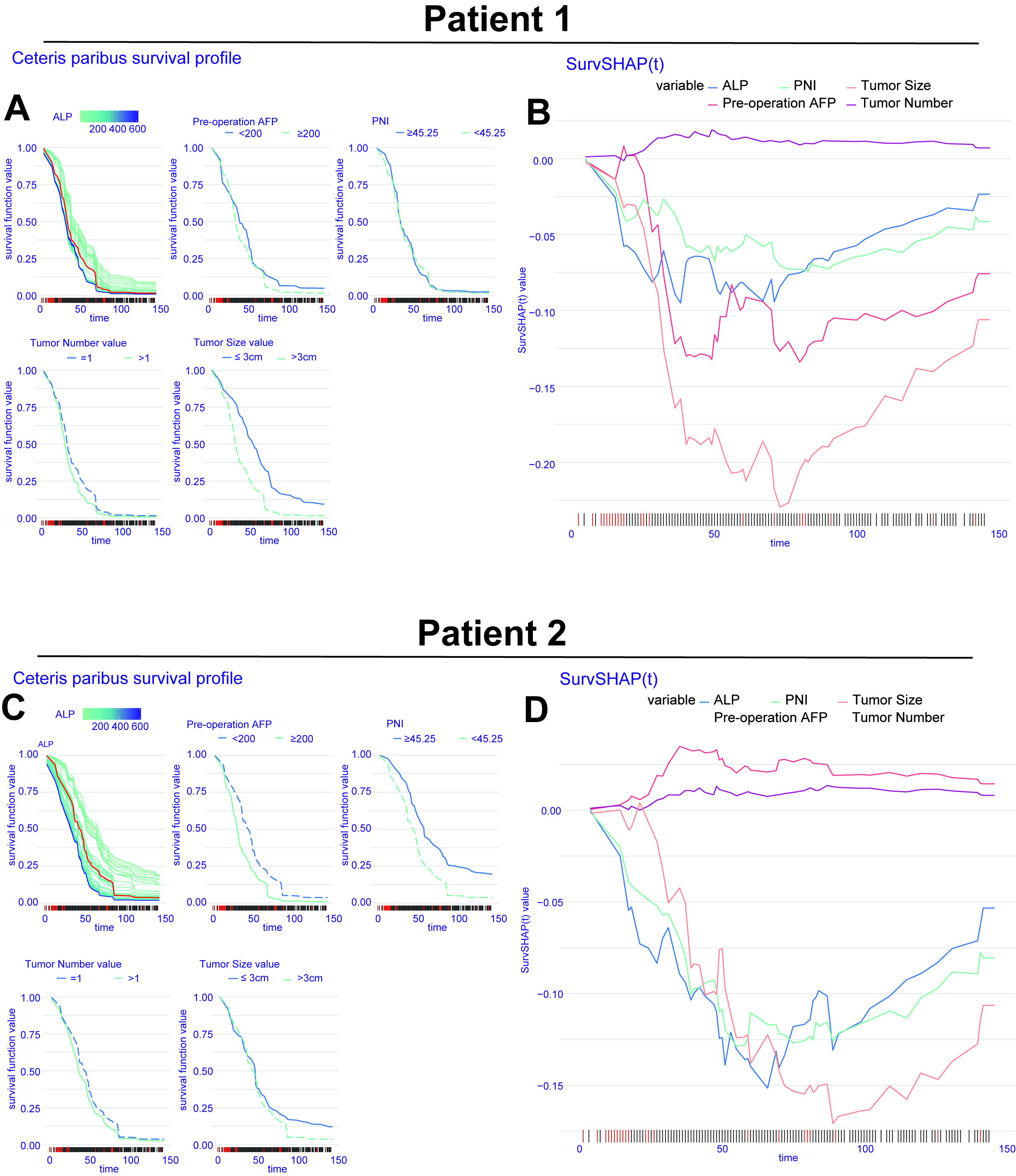

Supplement: Supplementary file 6 — Figure S6. [file CAM4-13-e70344-s001.tif]
