# Supplementary material for: Machine Learning Model Based on Prognostic Nutritional Index for Predicting Long‐Term Outcomes in Patients With HCC Undergoing Ablation
Source: Cancer Med. 2024 Oct 23;13(20):e70344. doi: 10.1002/cam4.70344 (PMC11496905; doi:10.1002/cam4.70344)

Figure S2 Univariable Cox regression analysis to identify predictors of overall survival in patients with HCC after ablation

Variable	Hazard ratio	pvalue
Sex (Male / Female)	0.928(0.653−1.320)	0.678
Age	1.020(1.010−1.030)	0.001
HBsAg (+ / -)	0.862(0.602−1.230)	0.419
HCVAb (+ / -)	1.110(0.636−1.950)	0.707
WBC	0.957(0.886−1.030)	0.267
NEU	0.990(0.900−1.090)	0.833
LYM	0.734(0.592−0.910)	0.005
ALP	1.010(1.000−1.010)	<0.001
Pre-operation AFP (<200 / ≥200)	2.050(1.550−2.730)	<0.001
ALT	1.000(0.998−1.010)	0.296
AST	1.000(1.000−1.010)	0.047
GGT	1.000(1.000−1.000)	<0.001
TBIL	1.020(1.010−1.040)	<0.001
ALB	0.926(0.902−0.952)	<0.001
PLT	0.998(0.996−1.000)	0.038
PT	1.240(1.130−1.350)	<0.001
PNI (<45.25 / ≥45.25)	2.080(1.610−2.680)	<0.001
HBV-DNA (+ / -)	0.959(0.724−1.270)	0.768
APRI	1.520(1.120−2.070)	0.007
Child-pugh (A / B)	2.190(1.270−3.760)	0.005
PS_score (0 / 1)	1.280(0.179−9.120)	0.807
Tumor Number (1 / >1)	1.630(1.190−2.240)	0.003
Tumor Size (≤3 / >3 cm)	1.910(1.450−2.520)	<0.001

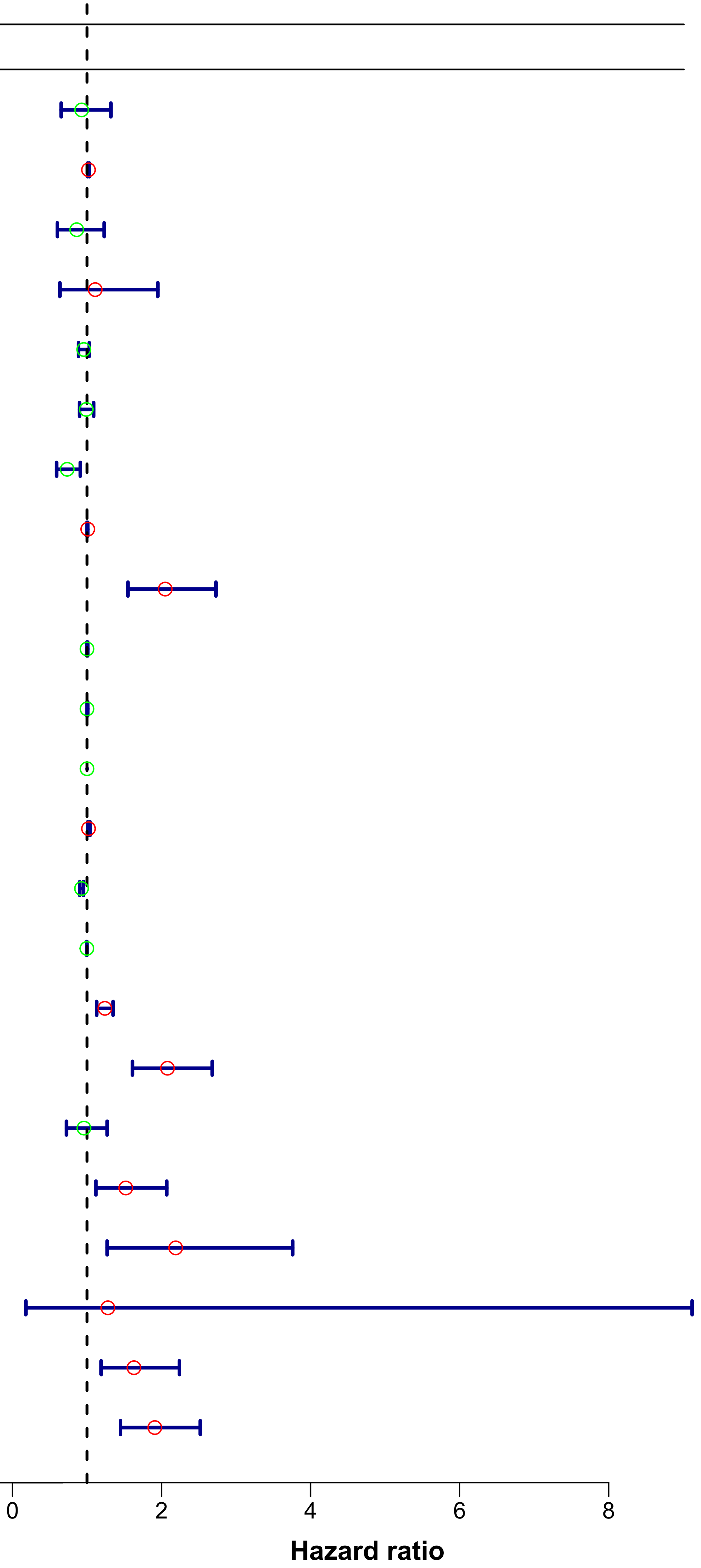

Supplement: Supplementary file 2 — Figure S2. [file CAM4-13-e70344-s005.pdf]
