# Supplementary material for: Machine Learning Model Based on Prognostic Nutritional Index for Predicting Long‐Term Outcomes in Patients With HCC Undergoing Ablation
Source: Cancer Med. 2024 Oct 23;13(20):e70344. doi: 10.1002/cam4.70344 (PMC11496905; doi:10.1002/cam4.70344)
Supplement: Supplementary file 7 — Data S1. [file CAM4-13-e70344-s004.docx]

**Machine learning model based on prognostic nutritional index for predicting long-term outcomes in patients with HCC undergoing ablation**

**Supplementary Materials**

1. **Supplementary Methods**

1.1 Details of machine learning models building

1.2 Ultrasound-guided percutaneous ablation procedure

1. **Supplementary Tables**

2.1 Table S1. Baseline characteristics of HCC patients stratified by PNI in different cohorts

1. **Supplementary Figures**

3.1 Figure S1. The log-rank test cutoff of PNI

3.2 Figure S2. Forest plot summary of univariate Cox analyses of prognosis.

3.3 Figure S3. Variable selection using Lasso regression

3.4 Figure S4. The C-index for 19 machine learning models

3.5 Figure S5. Performance of the Aorsf model in different subgroups

3.6 Figure S6. Individualized prediction using the SHAP interpretation of the Aorsf model

1. **Supplementary Methods
   1.1 Details of machine learning models building**

The codes and construction processes of machine learning were comprehensively documented in the GitHub repository hosted at https://github.com/mlr-org.

**1.2 Ultrasound-guided percutaneous ablation procedure**

The RFA or MWA procedures were performed percutaneously by one of the two radiologists, each with over 10 years of experience in tumor ablation. Conscious analgesia-sedation along with local anesthesia was administered during the ablation process. Ultrasound guidance was utilized for inserting either Cool-tip electrodes or Emprint MWA antenna into the target tumor. Ablation was carried out using predetermined power and duration parameters based on recommended protocols. In an effort to achieve complete tumor ablation with a safe margin of 10 mm, the electrodes were tracked back or repositioned as necessary. If an insufficient margin was detected on CEUS examination immediately after ablation, an additional ablation session would be attempted on the same day or following day after ablating the insertion tract.

**Supplementary Tables**

**2.1 Table S1 Baseline characteristics of HCC patients stratified by PNI in different cohorts**

Table S1 HBsAg, Hepatitis B surface antigen; HCVAb, Hepatitis C virus antibody; HBV-DNA, Hepatitis B virus DNA; WBC, White blood cell count, NEU, neutrophil; LYM, Lymphocyte; PLT, Platelet count; PNI, prognostic nutritional index; ALP, Alkaline phosphatase; ALT, alanine aminotransferase; AST, Aspartate aminotransferase; GGT, Gamma-glutamyl transferase; TBIL, Total bilirubin; ALB, Albumin; PT, Prothrombin time; APRI, Aspartate aminotransferase-to-platelet ratio index; ALBI, Albumin-bilirubin index; AFP, alpha fetoprotein; BCLC, Barcelona Clinic Liver Cancer; JIS, Japan Integrated Staging, CUPI, Chinese University Prognostic Index; CLIP, Cancer of the Liver Italian Program score; AJCC-TNM, American Joint Committee on Cancer tumor-node-metastasis staging system.

1. **Supplementary Figures**

**3.1** Figure S1 The determination of the log-rank test cutoff for PNI was performed, and the overall survival was assessed using the maximally selected log-rank test.

**3.2 Figure S2 Forest plot summary of univariate Cox analyses of prognosis.**

Univariate analyses were carried out using the clinical covariates in the training cohort based on overall survival. HBsAg, Hepatitis B surface antigen; HCVAb, Hepatitis C virus antibody; HBV-DNA, Hepatitis B virus DNA; WBC, White blood cell count, NEU, neutrophil; LYM, Lymphocyte; PLT, Platelet count; PNI, prognostic nutritional index; ALP, Alkaline phosphatase; ALT, alanine aminotransferase; AST, Aspartate aminotransferase; GGT, Gamma-glutamyl transferase; TBIL, Total bilirubin; ALB, Albumin; PT, Prothrombin time; APRI, Aspartate aminotransferase-to-platelet ratio index; AFP, alpha fetoprotein.

**3.3** Figure S3 A-B. The study examines the trajectories of coefficients for various independent variables and identifies the minimum error (lambda-min) as the determinant for 15 variables suitable for analysis in the follow-up study.

**3.4** Figure S4 The study reports the median and 95% confidence interval of the C-index for 19 machine learning models.

**3.5** Figure S5 Performance of the Aorsf model in different subgroups: A. Overall survival (OS) to Aorsf model in the training cohort subgroup with alpha-fetoprotein (AFP) levels <200μg/ml; B. OS to Aorsf model in the training cohort subgroup with a single tumor; C. OS to Aorsf model in the training cohort subgroup with tumor size ≤3cm; D. OS to Aorsf model in the validation cohort subgroup with AFP levels <200μg/ml; E. OS to Aorsf model in the validation cohort subgroup with a single tumor; F. OS to Aorsf model in the validation cohort subgroup with tumor size ≤3cm. PNI, prognostic nutritional index; AFP, alpha fetoprotein.

**3.6** Figure S6 Individualized prediction was performed on two patients using the SHAP interpretation of the Aorsf model to determine the impact of each feature on the model output (A, C). At each time point, each indicator showed a positive or negative distribution (B, D). The higher the curve, the higher the functional importance. PNI, prognostic nutritional index; ALP, Alkaline phosphatase; AFP, alpha fetoprotein.
