# Supplementary material for: Machine Learning Model Based on Prognostic Nutritional Index for Predicting Long‐Term Outcomes in Patients With HCC Undergoing Ablation
Source: Cancer Med. 2024 Oct 23;13(20):e70344. doi: 10.1002/cam4.70344 (PMC11496905; doi:10.1002/cam4.70344)
Supplement: Supplementary file 8 — Table S1. [file CAM4-13-e70344-s002.docx]

| **Table S1: Baseline characteristics of HCC patients stratified by PNI in different cohorts** | | | | | | | | | | | | | | |
| --- | --- | --- | --- | --- | --- | --- | --- | --- | --- | --- | --- | --- | --- | --- |
|  | |  | | **Training** | |  | |  | | **Validation** | |  | | |
|  | |  | | **(N=527)** | |  | |  | | **(N=224)** | |  | | |
|  | | **PNI≥45.25** | | **PNI<45.25** | | **P-value** | | **PNI≥43.2** | | **PNI<43.2** | | | **P-value** | |
|  |  | **(N=311)** | | **(N=216)** | |  |  | **(N=146)** | | **(N=78)** | | |  |  |
| **Age** | | 54.7 (11.9) | | 60.1 (10.6) | | <0.001 | | 54.5 (11.4) | | 60.7 (10.2) | | | <0.001 | |
| **ALB** | | 41.3 (3.57) | | 35.1 (4.34) | | <0.001 | | 41.1 (3.68) | | 34.6 (3.79) | | | <0.001 | |
| **ALP** | | 80.7 (26.1) | | 103 (55.4) | | <0.001 | | 82.5 (37.6) | | 95.0 (28.3) | | | 0.01 | |
| **ALT** | | 37.7 (27.6) | | 41.7 (32.0) | | 0.13 | | 36.0 (24.3) | | 41.2 (32.9) | | | 0.22 | |
| **APRI** | | 0.281 (0.282) | | 0.511 (0.411) | | <0.001 | | 0.269 (0.195) | | 0.557 (0.444) | | | <0.001 | |
| **AST** | | 36.7 (25.2) | | 45.8 (31.5) | | <0.001 | | 35.6 (18.0) | | 46.7 (35.5) | | | 0.01 | |
| **GGT** | | 65.9 (75.1) | | 85.9 (101) | | 0.01 | | 69.3 (97.2) | | 91.4 (87.7) | | | 0.09 | |
| **LYM** | | 2.05 (0.624) | | 1.36 (0.412) | | <0.001 | | 1.87 (0.656) | | 1.29 (0.352) | | | <0.001 | |
| **NEU** | | 3.21 (1.32) | | 2.76 (1.55) | | <0.001 | | 3.31 (1.60) | | 2.60 (1.64) | | | 0.00 | |
| **PLT** | | 166 (70.0) | | 116 (62.3) | | <0.001 | | 160 (67.4) | | 110 (66.7) | | | <0.001 | |
| **PT** | | 12.3 (1.14) | | 13.4 (1.44) | | <0.001 | | 12.5 (1.12) | | 13.5 (1.30) | | | <0.001 | |
| **TBIL** | | 15.6 (7.29) | | 20.6 (10.9) | | <0.001 | | 14.8 (7.11) | | 20.4 (11.0) | | | <0.001 | |
| **WBC** | | 5.99 (1.62) | | 4.86 (1.84) | | <0.001 | | 5.88 (1.85) | | 4.49 (1.77) | | | <0.001 | |
| **Child-pugh** | |  | |  | |  | |  | |  | | |  | |
| **Stage A** | | 311 (100%) | | 194 (89.8%) | | <0.001 | | 143 (97.9%) | | 66 (84.6%) | | | <0.001 | |
| **Stage B** | | 0 (0%) | | 22 (10.2%) | |  | | 3 (2.1%) | | 12 (15.4%) | | |  | |
| **HBsAg** | |  | |  | |  | |  | |  | | |  | |
| **-** | | 47 (15.1%) | | 32 (14.8%) | | 1.00 | | 22 (15.1%) | | 9 (11.5%) | | | 0.60 | |
| **+** | | 264 (84.9%) | | 184 (85.2%) | | | | 124 (84.9%) | | 69 (88.5%) | | |  | |
| **HBV-DNA** | |  | |  | |  | |  | |  | | |  | |
| **<100×10^2^** | | 202 (65.0%) | | 148 (68.5%) | | 0.45 | | 93 (63.7%) | | 49 (62.8%) | | | 1.00 | |
| **≥100×10^2^** | | 109 (35.0%) | | 68 (31.5%) | |  | | 53 (36.3%) | | 29 (37.2%) | | |  | |
| **HCVAb** | |  | |  | |  | |  | |  | | |  | |
| **-** | | 295 (94.9%) | | 202 (93.5%) | | 0.65 | | 143 (97.9%) | | 75 (96.2%) | | | 0.72 | |
| **+** | | 16 (5.1%) | | 14 (6.5%) | |  | | 3 (2.1%) | | 3 (3.8%) | | |  | |
| **Pre-operation AFP** | | | |  | |  | |  | |  | | |  | |
| **<200** | | 259 (83.3%) | | 164 (75.9%) | | 0.05 | | 121 (82.9%) | | 62 (79.5%) | | | 0.66 | |
| **≥200** | | 52 (16.7%) | | 52 (24.1%) | |  | | 25 (17.1%) | | 16 (20.5%) | | |  | |
| **PS_score** | |  | |  | |  | |  | |  | | |  | |
| **0** | | 310 (99.7%) | | 215 (99.5%) | | 1.00 | | 143 (97.9%) | | 77 (98.7%) | | | 1.00 | |
| **1** | | 1 (0.3%) | | 1 (0.5%) | |  | | 3 (2.1%) | | 1 (1.3%) | | |  | |
| **Sex** | |  | |  | |  | |  | |  | | |  | |
| **Male** | | 266 (85.5%) | | 182 (84.3%) | | 0.78 | | 125 (85.6%) | | 66 (84.6%) | | | 1.00 | |
| **Female** | | 45 (14.5%) | | 34 (15.7%) | |  | | 21 (14.4%) | | 12 (15.4%) | | |  | |
| **Tumor Number** | | | |  | |  | |  | |  | | |  | |
| **1** | | 270 (86.8%) | | 172 (79.6%) | | 0.04 | | 130 (89.0%) | | 65 (83.3%) | | | 0.32 | |
| **>1** | | 41 (13.2%) | | 44 (20.4%) | |  | | 16 (11.0%) | | 13 (16.7%) | | |  | |
| **Tumor Size** | | | |  | |  | |  | |  | | |  | |
| **≤3cm** | | 242 (77.8%) | | 169 (78.2%) | | 0.99 | | 113 (77.4%) | | 57 (73.1%) | | | 0.58 | |
| **>3cm** | | 69 (22.2%) | | 47 (21.8%) | |  | | 33 (22.6%) | | 21 (26.9%) | | |  | |
| **BCLC** | |  | |  | |  | |  | |  | | |  | |
| **0** | | 102 (32.8%) | | 54 (25.0%) | | 0.07 | | 55 (37.7%) | | 18 (23.1%) | | | 0.04 | |
| **A** | | 209 (67.2%) | | 162 (75.0%) | | | | 91 (62.3%) | | 60 (76.9%) | | |  | |
| **CLIP** | |  | |  | |  | |  | |  | | |  | |
| **0** | | 240 (77.2%) | | 131 (60.6%) | | <0.001 | | 108 (74.0%) | | 47 (60.3%) | | | 0.02 | |
| **1** | | 66 (21.2%) | | 70 (32.4%) | |  | | 37 (25.3%) | | 27 (34.6%) | | |  | |
| **2** | | 5 (1.6%) | | 15 (6.9%) | |  | | 1 (0.7%) | | 4 (5.1%) | | |  | |
| **TNM** | |  | |  | |  | |  | |  | | |  | |
| **T1a** | | 102 (32.8%) | | 54 (25.0%) | | 0.03 | | 55 (37.7%) | | 18 (23.1%) | | | 0.07 | |
| **T1b** | | 168 (54.0%) | | 118 (54.6%) | | | | 75 (51.4%) | | 47 (60.3%) | | |  | |
| **T2** | | 41 (13.2%) | | 44 (20.4%) | |  | | 16 (11.0%) | | 13 (16.7%) | | |  | |
| **French** | |  | |  | |  | |  | |  | | |  | |
| **0** | | 270 (86.8%) | | 172 (79.6%) | | 0.04 | | 130 (89.0%) | | 65 (83.3%) | | | 0.32 | |
| **1** | | 41 (13.2%) | | 44 (20.4%) | |  | | 16 (11.0%) | | 13 (16.7%) | | |  | |
| **CUPI** | |  | |  | |  | |  | |  | | |  | |
| **Low risk** | | 310 (99.7%) | | 203 (94.0%) | | <0.001 | | 146 (100%) | | 73 (93.6%) | | | 0.01 | |
| **Intermediate risk** | | 1 (0.3%) | | 13 (6.0%) | |  | | 0 (0%) | | 5 (6.4%) | | |  | |
| **JIS** | |  | |  | |  | |  | |  | | |  | |
| **0** | | 270 (86.8%) | | 172 (79.6%) | | 0.04 | | 130 (89.0%) | | 65 (83.3%) | | | 0.32 | |
| **1** | | 41 (13.2%) | | 44 (20.4%) | |  | | 16 (11.0%) | | 13 (16.7%) | | |  | |
| **Okuda** | |  | |  | |  | |  | |  | | |  | |
| **Stage I** | | 270 (86.8%) | | 172 (79.6%) | | 0.04 | | 130 (89.0%) | | 65 (83.3%) | | | 0.32 | |
| **Stage II** | | 41 (13.2%) | | 44 (20.4%) | |  | | 16 (11.0%) | | 13 (16.7%) | | |  | |
|  | |  | |  | |  | |  | |  | |  | | |
